# Stepped-wedge cluster randomised trials: where, when and why?

**DOI:** 10.1186/1745-6215-16-S2-P132

**Published:** 2015-11-16

**Authors:** Michael Grayling, James Wason, Adrian Mander

**Affiliations:** 1 MRC Biostatistics Unit, Cambridge, UK

## 

The stepped wedge (SW) cluster randomised trial (CRT) design is being utilised at increasing pace. However, little is known about the standard of reporting of such trials, or how useful the design has proven to be in hindsight. Moreover, much debate exists around when the design should be preferred to the more classical parallel group (PG) CRT. Here, we address these issues by first conducting a thorough review of all SW-CRTs. We are able to ascertain not only the quality of reporting, but also the stated reasons for the design's use. We are also able to highlight instances where, on reflection, alternative designs may have been preferable. We then present a critical appraisal of the design from a logistical and ethical standpoint. With our findings we propose methodology for the incorporation of early stopping for futility within a SW-CRT. Finally, we compare this new design in terms of expected efficiency to the conventional approach as well as to several variants of the PG-CRT design. We assess which is optimal in a range of settings, including balancing the required sample size with the required time for trial completion. We find that to date the standard of reporting of SW-CRTs has been mixed in quality. However, there are many instances in which the design is preferable to the PG-CRT approach. In particular, through the addition of early futility stopping, sample size savings under the null hypothesis of more than 30% can be observed, at little cost to the length of the trial.

